# CSF Metabolic and Proteomic Profiles in Patients Prodromal for Psychosis

**DOI:** 10.1371/journal.pone.0000756

**Published:** 2007-08-22

**Authors:** Jeffrey T.-J. Huang, F. Markus Leweke, Tsz M. Tsang, Dagmar Koethe, Laura Kranaster, Christoph W. Gerth, Sonja Gross, Daniela Schreiber, Stephan Ruhrmann, Frauke Schultze-Lutter, Joachim Klosterkötter, Elaine Holmes, Sabine Bahn

**Affiliations:** 1 Institute of Biotechnology, University of Cambridge, Cambridge, United Kingdom; 2 Department of Psychiatry and Psychotherapy, University of Cologne, Cologne, Germany; 3 Department of Biomolecular Medicine, Division of SORA, Faculty of Medicine, Imperial College, London, United Kingdom; James Cook University, Australia

## Abstract

**Background:**

The initial prodromal state of psychosis (IPS) is defined as an early disease stage prior to the onset of overt psychosis characterized by sub-threshold or more unspecific psychiatric symptoms. Little is known regarding the biochemical changes during this period.

**Methodology/Principal Findings:**

We investigated the metabolic/proteomic profiles of cerebrospinal fluid (CSF) of first-onset drug naïve paranoid schizophrenia patients (n = 54) and individuals presenting with initial prodromal symptoms (n = 24), alongside healthy volunteers (n = 70) using proton nuclear magnetic resonance (^1^H-NMR) spectroscopy and surface enhanced laser desorption ionization (SELDI) mass spectrometry, respectively. Partial least square discriminant analysis (PLS-DA) showed that 36%/29% of IPS patients displayed proteomic/metabolic profiles characteristic of first-onset, drug naïve schizophrenia, i.e., changes in levels of glucose and lactate as well as changes in a VGF-derived peptide (VGF23-62) and transthyretin protein concentrations. However, only 29% (n = 7) of the investigated IPS patients (who to date have been followed up for up to three years) have so far received a diagnosis of schizophrenia. The presence of biochemical alterations in the IPS group did not correlate with the risk to develop schizophrenia.

**Conclusions/Significance:**

Our results imply that schizophrenia-related biochemical disease processes can be traced in CSF of prodromal patients. However, the biochemical disturbances identified in IPS patients, at least when measured at a single time point, may not be sufficient to predict clinical outcome.

## Introduction

Prior to the onset of frank psychosis, at-risk patients usually present with non-specific signs and symptoms, including reduced concentration and motivation, depressed mood, sleep disturbances, anxiety, social withdrawal, suspiciousness, deterioration in social functioning, and increased irritability [1]. The duration of the initial prodromal state of psychosis (IPS) is extremely variable, ranging from a few weeks to several years and is often indistinguishable from more severe adolescent behavioural problems. The initial prodrome may thus reflect a vulnerable period in which deleterious environmental factors may precipitate the transition from IPS to the onset of overt psychosis and in turn schizophrenia. This period provides a window of opportunity for gaining a better understanding of the pathological process of schizophrenic psychosis as well as early diagnosis and intervention; especially as strong evidence suggests that early treatment is associated with a better prognosis [2], [3].

Most efforts to elucidate the IPS have to date focused on the identification of symptomatic alterations in IPS patients to predict outcome [4]–[6]. In our recent studies on first onset, drug naïve paranoid schizophrenia patients we identified significant alterations in the metabolic and proteomic profiles in CSF [7], [8]. The key metabolic changes included elevated glucose levels, reduced lactate and acetate levels and a decrease in pH [8]. We also found increased levels of a VGF-derived peptide (VGF23-62 fragment) and a decrease in transthyretin protein concentrations in our proteomic profiling studies [7]. In the present study, we investigated whether the same or similar CSF alterations can also be traced in patients meeting symptomatic criteria of an IPS.

## Results

We employed ^1^H-NMR spectroscopy-based metabonomics and SELDI mass spectrometry-based proteomics techniques to investigate the CSF profiles of 24 patients in IPS alongside 54 first-onset drug naive schizophrenia patients and 70 matched healthy volunteers (Table 1). Results of the comparison of first-onset schizophrenia patients and controls have been published elsewhere [7], [8].

**Table 1:** Demographic details of subjects in the metabonomics study

	Healthy volunteers (n = 70)	First-onset, drug naïve schizophrenia patients (n = 49)	IPS patients (n = 24)
**Age (yrs)** *	27.4±5.9#	27.2±8.6	24.2±5.0
**Gender^&^**			
** male**	39	39	18
** female**	31	15	6

# There is no significant difference in age between the control and disease groups.

* Data are shown in Mean±SD

**Figure 1:**
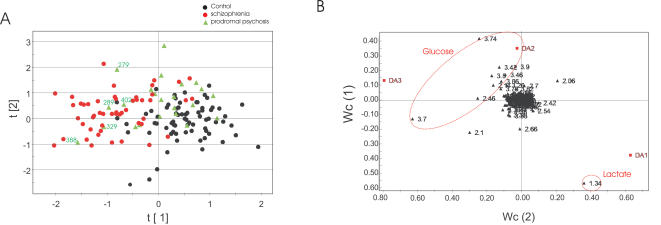
Metabonomic analysis of CSF samples from control subjects, patients suffering an IPS, and first onset, drug-naïve schizophrenic patients. (A) and (B) PLS-DA scores and variable loadings plots showing a clear separation of CSF samples derived from drug-naïve schizophrenia patients (red dots) compared to demographically matched controls (black dots) as determined by the ^1^H-NMR spectroscopy IPS patients are shown as green triangles. 33% (n = 8) of the IPS patients were found to cluster with the schizophrenia patients (showing similar metabolic alterations). The key changing metabolic signals are from lactate and glucose resonances as well as a shift in glutamine. The id's of the five IPS patients who showed the greatest metabolic disturbances are displayed on the plot. We found that the presence of metabolic disturbances was highly predictive of the presence of protein abnormalities (see Fig 2).

When comparing the metabolic profiles using PLS-DA, we observed that IPS patients were distributed across, but mainly in-between, the distinct clusters of first-onset drug naïve schizophrenia patients and healthy controls (Figure 1A). The key differentiating metabolites were CSF glucose and lactate levels (Figure 1B). 33% of IPS patients (n = 8) co-clustered with the schizophrenia group and 50% (n = 12) co-clustered with controls. When investigating the whole IPS group (n = 24), neither the comparison between prodromal patients and controls, nor the comparison between prodromal and first-onset, drug naïve schizophrenia patients showed a significant difference (data not shown).

**Figure 2:**
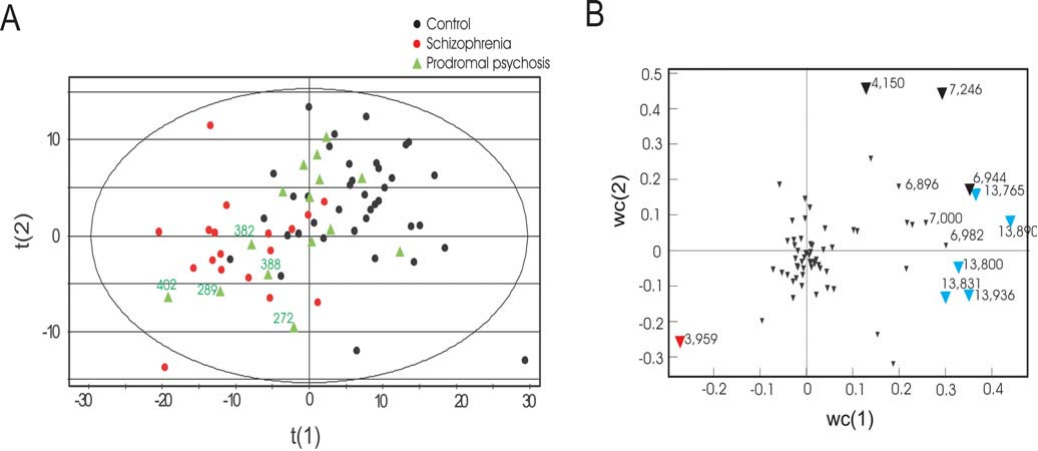
Proteomic analyses of CSF samples from control subjects, patients suffering an IPS, and first onset, drug-naïve schizophrenic patients. (A) and (B) PLS-DA scores and loadings plot showing a differentiation of drug-naïve schizophrenia patients (red dots) from demographically matched controls (black dots) as determined by SELDI-MS spectra. IPS patients are shown in green triangles. 33% of the IPS patients were found to cluster with the schizophrenia patients (showing similar protein alterations). The key changing proteins are a VGF23-62 peptide (at m/z = 3,959, red inverted triangle) and transthyretin proteins (m/z = 13.7−14.0k; blue inverted triangle). The id's of the six IPS patients who showed the greatest protein disturbances are marked/numbered on the plot. We found that the presence of protein disturbances was highly predictive of the presence of metabolite abnormalities (see Fig 1).

**Figure 3:**
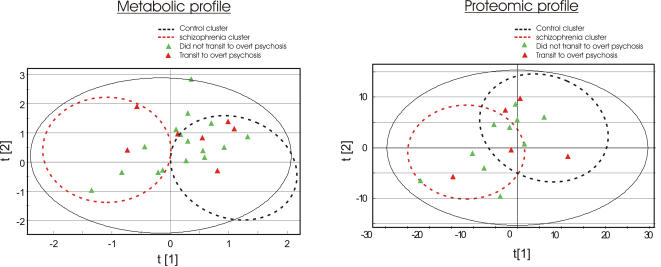
No clear association was found between clinical outcomes and metabolic/proteomic profiles of patients suffering an IPS. CSF metabolic and proteomic profiles of IPS patients were analyzed by ^1^H-NMR spectroscopy and SELDI-MS, respectively. PLS models based on CSF profiles from controls and first onset schizophrenia patients were built to try to predict the transition of IPS patients to develop overt psychosis. IPS patients were followed up for up to 3 years. Those IPS patients who went on to develop overt psychosis are labelled in red. There is no clear association between clinical outcomes and their CSF metabolic (Figure 3A) and proteomic (Figure 3B) profiles at the prodromal phase of psychosis.

Similarly, we investigated CSF proteomic profiles in 14 IPS patients, 17 first-onset, drug naïve schizophrenia patients and 40 healthy volunteers using SELDI mass spectrometry (Table 2). CSF proteomic profiles of prodromal patients again were distributed across the clusters of controls and schizophrenia patients (Figure 2A). Among the IPS patients, 36% (n = 5) co-clustered with the schizophrenia group. The most important factors contributing to the partial separation between schizophrenia patients and controls were the VGF23-62 peptide (at m/z = 3,959) and transthyretin proteins (at m/z = 13.7−14.0k) (Figure 2B). Again, these differences were not statistically significant when the IPS group was compared to the control or schizophrenia groups, respectively. Interestingly, we found that the 5 IPS patients whose proteomic profiles showed schizophrenia-like changes, also showed significant schizophrenia-like metabolic alterations (Figure 1 and 2). To date IPS patients have been followed up for three years and 29 % (n = 7) of the 24 IPS patients have developed schizophrenia within this period. We examined whether the presence of schizophrenia-like CSF metabolic and proteomic alterations in prodromal patients could predict a subsequent transition to overt schizophrenia. However, we did not observe a correlation between abnormalities in CSF profiles and subsequent transition to overt schizophrenia (Figure 3).

**Table 2:** Demographic details of subjects in the SELDI study

	Healthy volunteers (n = 40)	First-onset, drug naïve schizophrenia patients (n = 17)	IPS patients (n = 14)
**Age (yrs)** *	27.3±3.8	27.6±7.9	23.9±5.1
**Gender**			
** male**	20	10	10
** female**	20	7	4

* Data are shown as Mean±SD

Based on the above results, we propose that CSF metabolic and proteomic changes in the initial prodromal state of psychosis reflect a molecular disturbance associated with psychosis. However, the biochemical investigation of CSF at a single time point during the initial prodrome may not be sufficient to predict clinical outcome, possibly due to fluctuations/variation of CSF metabolic/proteomic profiles during the prodromal phase. Future longitudinal studies with multiple assessment times are required to test this notion.

## Discussion

**Figure 4:**
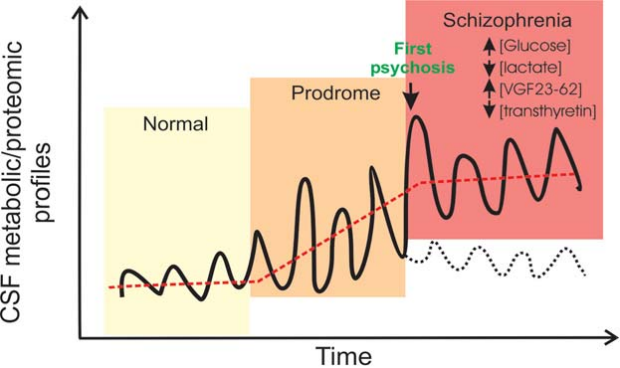
A hypothetical model of alterations in the CSF metabonome and proteome observed in the IPS and in the schizophrenia phase. In the schizophrenia phase, patients have a distinct metabolic and protein/peptide profiles in CSF, i.e. increased glucose and VGF23-62 peptide levels and reduced lactate and transthyretin protein levels. During the IPS, key biochemical alterations may vary possibly in accordance with “mental-state” fluctuations between mental well being and schizophrenia-like states. During the first episode of overt psychosis, these alterations become more persistent and significant. For some IPS patients, their conditions may not deteriorate and their CSF proteomic and metabolic profiles may return to normal.

IPS may be a critical window of opportunity for the prevention of schizophrenia. In the past, studies on IPS patients have predominantly focused on the identification of psychopathological as well as, though to a lesser degree, neurocognitive and structural cerebral changes (e.g.[1], [5], [9]–[13]). The present study is the first attempt to investigate the biochemical processes associated with the IPS. The specific interest of the present study was to explore whether schizophrenia-related biomarkers can be identified in IPS patients and, if this was the case, whether a biomarker signature can predict the transition from IPS to overt psychosis. Whilst we found significant changes between controls and first-onset schizophrenia patients in CSF samples [7], [8], no significant differences were found when comparing IPS patients to controls, and IPS patients to schizophrenia patients. We found that the proteomic and metabolic profiles of IPS patients overlapped substantially with the profiles of healthy volunteers as well as first-onset schizophrenia patients. This is conceptually not surprising, as symptoms during the IPS are less persistent and specific (and may even return to “normal”), which could be reflected in the disease-related biochemical read-outs (Figure 4). These data imply that metabolic and proteomic alterations in CSF, i.e. increased glucose and VGF23-62 peptide levels and reduced lactate and transthyretin levels, are becoming more significant and persistent during the first episode of overt psychosis.

The pathological significance of the metabolic and proteomic alterations observed in CSF in first-onset schizophrenia and approx. 33% of the IPS patients require further investigation. As the increase in glucose levels coincides with a decrease in lactate levels in schizophrenia CSF, a possible scenario is that the diseased brain utilizes lactate (which is predominantly produced by astrocytes [14]) instead of glucose as a primary energy source. This has been suggested in a number of pathological conditions such as diabetes and prolonged starvation [15], [16]. With regards to the protein/peptide alterations, the increased levels of the VGF23-62 peptide may indicate an increase in the native (full-length) VGF protein and/or abnormalities in the processing of the protein within the brain. Indeed, our previous study showed that VGF protein expression was increased in half of the investigated schizophrenia post-mortem brains [7]. VGF is a secreted polypeptide that is selectively expressed by neurons and several endocrine and neuroendocrine tissues (for review [17], [18]), and is transported in dense core vesicles with the VGF-derived processed peptides being released via a regulated secretory pathway [17], [18]. Knockout of the VGF gene in mice generated a lean, hypermetabolic, hyperactive phenotype, demonstrating that VGF may regulate energy balance [19]. The observation of an increase of the VGF peptide in CSF from schizophrenia patients, therefore, may point to a hypometabolic state in the schizophrenia brain. On the other hand, as transthyretin is a thyroid hormone-binding protein that transports thyroxine from the bloodstream to the brain [20], the decreased level of transthyretin in CSF could imply abnormalities in thyroxine transport in brains of schizophrenia patients. Taken together, our observations indicate that in first onset schizophrenia (as well as about 33% of the prodromal patients), the brain may be in a hypo-metabolic state and that the preferred energy source may be switched to lactate instead of glucose. Interestingly, a large scale investigation on long-term antipsychotic treated rats in our laboratory showed that several antipsychotic drugs increase brain lactate levels (manuscript in preparation).

A particular interest of the present study was to explore whether prodromal patients who co-clustered with the schizophrenia group with regards to their CSF proteomic and metabolic profiles have a higher risk of developing schizophrenia. Unfortunately, we did not find an association between the presence of biochemical changes in IPS patients and the eventual transition to schizophrenia. There are several possible explanations for our observations: (1). CSF proteomic and metabolic profiles may fluctuate depending on mental/physical state. Thus, measurements from a single time point (as investigated in this study) are not sufficient. Multiple measurements may have better prediction power. (2). Predictive biomarkers may be expressed at very low concentration and the technologies used in this study may not have been sensitive enough. Applying more sensitive methods such as LC-MS/MS may provide a solution. (3). Changes in CSF in the IPS may not be indicative of the risk of developing schizophrenia, though they may still correlate with the pathological process of psychosis.

Of the 24 IPS patients included in this study, 29% (n = 7) developed overt psychosis/schizophrenia within a 3 year follow-up period in the current study. This observed transition rate is lower compared to two previous studies. The Cologne Early Recognition – CER study [4], ascertained an initial cohort of 385 patients with symptoms of initially prodromal schizophrenia. Of these 160 patients were re-assessed within a mean period of 9.6 years of the initial assessment. 65% of the 160 patients had developed schizophrenic psychosis-20 % within one year of the baseline assessment, 17% within two and 13% within three years, whereas the transition rate in those who did not report any of these symptoms was only 18%. Based on the “ultra high risk” (UHR) criteria of imminent risk of psychosis, the average 12-months transition rate in untreated patients is 38%, with not much knowledge as to the rate of transition in the following years [6].

In summary, we found similar alterations in the metabolic and proteomic CSF profiles in a subgroup of initially prodromal and first-onset schizophrenia patients. Our results suggest a progression of distinct biochemical disturbances, which may be intermittent in the prodromal disease phase. However, as the number of IPS patients who transgressed to overt psychosis is at present small, further studies are required. A preferred (and less invasive) approach would be to identify serum biomarkers for early/prodromal diagnosis; in that case multiple sampling to increase the chance of correctly identifying a fluctuating biochemical perturbation is clinically feasible.

## Methods and Materials

### Clinical samples

The Ethical Committee of the Medical Faculty of the University of Cologne reviewed and approved the protocol of this study and the procedures for sample collection and analysis. All study participants gave their written informed consent. All clinical investigations were conducted according to the principles expressed in the Declaration of Helsinki. CSF samples were collected from prodromal patients meeting criteria for initial prodromal state of psychosis (IPS) (n = 24) as described previously [10], [21], drug-naïve patients diagnosed with first-onset paranoid schizophrenia or brief psychotic disorder due to duration of illness (DSM-IV 295.30, n = 54) and from demographically matched healthy volunteers (n = 70) (Table 1).

All samples were collected in a standardized fashion by the same team of experienced clinicians using a non-traumatic lumbar puncture procedure [22]. Trained clinical psychiatrists performed clinical assessments. Glucose levels in CSF and serum from healthy subjects, IPS and schizophrenic patients were measured immediately after collection using a NOVA BioProfile analyzer (Nova Biomedical, Waltham, USA). CSF samples were divided into aliquots and stored at −80°C. None of the samples underwent more than 2 freeze-thaw cycles prior to experiments. All experiments were performed under blind and randomized conditions.

### Diagnosis and inclusion/exclusion criteria for initial prodromal patients

Patients diagnosed with an IPS (n = 24) had sought help for mental health problems at the Cologne Early Recognition and Intervention Centre (FETZ), University of Cologne, and had been admitted to hospital for diagnostic reasons. An IPS was assumed if one or more of ten basic cognitive-perceptive symptoms was present, which were found to be highly predictive of schizophrenia in an earlier prospective study [4]. Or, in line with the so-called ‘UHR’ criteria [6], if attenuated (APS) or transient psychotic symptoms were present. The UHR criteria, aiming to detect imminent risk of psychosis, have shown an average 12-month conversion rate of 38.2% in non-treated prodromal subjects predominantly with APS [6], [11], [23]–[26]. The ten basic symptoms were included as an extension to these UHR criteria to identify an even earlier state of the initial prodrome. A prospective study has shown that 20% of patients presenting with one or more of the ten basic symptoms had developed schizophrenia within the first year, an additional 17% in the second, 13% in the third, and 15% within 4 to 14 years after first examination [4], [5].

Inclusion criteria for an IPS were: (i) One or more of the following basic symptoms with a score of 3 or more on the SPI-A [27] which had first presented at least one year ago: thought interferences; thought perseveration; thought pressure; thought blockages; disturbances of receptive language, either heard or read; decreased ability to discriminate between ideas and perception, fantasy and true memories; unstable ideas of reference; derealization; visual and/or acoustic perception disturbances; (ii) one or more of the following APS with a score of 3 to 5 on SOPS [10] within the last three months, appearing several times per week for a period of at least one week and, with the same severity, for no longer than one year: ideas of reference; odd beliefs or magical thinking; unusual perceptual experiences; odd thinking and speech; suspiciousness or paranoid ideation; (iii) one or more of the following brief limited intermittent psychotic symptoms with a PANSS score of at least 4 [6] for less than one week and an interval between episodes at least one week, resolving spontaneously: hallucinations; delusions; formal thought disorder; gross disorganized or catatonic behaviour.

Exclusion criteria for an IPS were: (i) present or past psychotic episode; (ii) current substance abuse or dependence; (iii) neurological, cerebral or other somatic illness that may account for the symptoms; (iv) mental retardation, and (v) age below 18 or above 40 years.

Prodromal symptoms were assessed by trained interviewers using the Structured Interview for the Scale of Prodromal Syndromes (SIPS/SOPS [28]), the Positive and Negative Syndrome Scale (PANSS [21]) and the Schizophrenia Proneness Instrument-Adult version (SPI-A [27]). The SIPS/SOPS had a pair-wise interrater concordance of 77% between four raters, the PANSS 82%, and the SPI-A had a concordance rate with an expert rating of 92%. The German version of the Structured Clinical Interview for DSM-IV [29] was administered to ascertain exclusion criteria disorders.

Altogether 7 (29.1%) of the IPS patients have meanwhile made the transition to overt psychosis, 4 to schizophrenia, paranoid subtype, 1 to schizophrenia, undifferentiated subtype, 1 to schizoaffective disorder and 1 to delusional disorder. The mean time from baseline assessment to transition was 14.3±5.3 months. All not yet converted patients were followed up for at least three years.

### ^1^H-NMR Spectroscopy of CSF Samples

The volume of CSF samples (150 µl) was made up to a final volume of 500 µl by the addition of D_2_O in preparation for ^1^H-NMR spectroscopic analysis. Standard 1-D 600 MHz ^1^H-NMR spectra were acquired for all samples using a pre-saturation pulse sequence to effect suppression of the water resonance (pulse sequence: relaxation delay-90°-t_1_-90°-t_m_-90°-acquire FID; Bruker Analytische GmbH, Rheinstetten, Germany). In this pulse sequence, a secondary radio frequency irradiation is applied specifically at the water resonance frequency during the relaxation delay of 2s and the mixing period (t_m_ = 100 ms), with t_1_ fixed at 3 µs. Typically 256 transients were acquired at 300K into 32K data points, with a spectral width of 6000Hz and an acquisition time of 1.36s per scan. Prior to Fourier transformation, the free induction decays were multiplied by an exponential weight function corresponding to a line-broadening factor of 0.3 Hz.

### Preparation of CSF Samples for SELDI Analysis

5 µl of each CSF sample was applied to protein chips with different chemical properties at various pH conditions. The best condition was chosen at pH9.0 on strong anion exchanger Q10 chip, based on number and separation of peaks resolved. Briefly, the array spots were pre-activated twice with binding buffer (100mM Tris-HCl, pH 9.0) at room temperature for 10 min on a shaker (frequency = 600 rpm). 50 µl binding buffer was added into each spot prior to the addition of 5 µl CSF sample. The protein chips were incubated on a shaker for 60 min at room temperature, then washed twice with binding buffer, once with H_2_O, and air-dried. The chips were then sequentially treated twice with 0.6 µl of a 100% saturated sinapinic acid (3, 5-dimethoxy-4-hydroxycinnamic acid) in 50% acetonitrile and 0.5% trifluoroacetic acid. The chips were analyzed with the Bio-Rad ProteinChip Reader (Bio-Rad ProteinChip System Series 4000). Each sample was analyzed twice to confirm reproducibility in identifying the differentially expressed proteins and to ensure stability of instrument performance.

### SELDI-TOF-MS Analysis

The arrays were analyzed with the Bio-Rad ProteinChip System Series 4000 (Bio-Rad, USA). Mass spectra of proteins were generated by using an average of 254 laser shots at a laser intensity of 2000 (nJ). For data acquisition, the detection size range was between 2.5 and 200 kDa. The laser was focused at 10 kDa. The mass-to-charge ratio (*m*/*z*) of each of the proteins captured on the array surface was determined relative to external calibration standards (Bio-Rad; USA): bovine insulin (5,733.6 Da), human ubiquitin (8,564.8 Da), bovine cytochrome *c* (12,230.9 Da), bovine superoxide dismutase (15,591.4 Da), horseradish peroxidase (43,240 Da) and BSA (66,410 Da). The data were analyzed with PROTEINCHIP data analysis software version 3.0 and Ciphergen Express Software 3.0 (Bio-Rad; USA). Matrix attenuation was set at 2,500Da and shot sequence was set with one warming shot at 2000 (nJ) followed by 4 data shot at 1800 (nJ). The Ciphergen Express Software 3.0 was used to compile all spectra and autodetect quantified mass peaks within a mass range between 2,500–200,000 Da and signal to noise threshold was set at 5. Peak labelling was completed by using second-pass peak selection with 0.2% of the mass window, and estimated peaks were added. The peak information of all spectra was exported for further statistic analyses.

### Data Reduction and Pattern Recognition Procedures

To efficiently evaluate the metabolic variability within and between biofluids derived from patients and controls, spectra were data reduced using the software program AMIX (Analysis of MIXtures version 2.5, Bruker Rheinstetten, Germany) and exported into SIMCA-P (Version 11.0, Umetrics AB, Umeå, Sweden) where a range of multivariate statistical analyses were conducted. For proteomic studies, the readings from each peak with S/N>5 were output and exported into SIMCA-P for further analysis. Initially principal components analysis was applied to the data in order to discern the presence of inherent similarities in spectral profiles. Where the classification of ^1^H-NMR spectra was influenced by exogenous contaminants, the spectral regions containing those signals were removed from statistical analysis. In order to confirm the biomarkers differentiating between the schizophrenia patients and matched controls, projection to PLS-DA was employed.

## Supporting Information

Figure S1 Proteomic analysis of CSF samples from patients with prodromal schizophrenia, and depression patients. (A) and (B) PLS-DA scores plots showing a degree of separation of prodromal schizophrenia patients (▪) from depression patients (•) as determined by the SELDI CSF spectra. The key changing proteomic peaks are from secretogranin II (529-566) and two transthyretin isoforms.
(0.99 MB DOC)
