# Supplementary material for: CSF Metabolic and Proteomic Profiles in Patients Prodromal for Psychosis
Source: PLoS One. 2007 Aug 22;2(8):e756. doi: 10.1371/journal.pone.0000756 (PMC1942084; doi:10.1371/journal.pone.0000756)
Supplement: Figure S1 — Proteomic analysis of CSF samples from patients with prodromal schizophrenia, and depression patients. (A) and (B) PLS-DA scores plots showing a degree of separation of prodromal schizophrenia patients (▪) from depression patients (•) as determined by the SELDI CSF spectra. The key changing proteomic peaks are from secretogranin II (529-566) and two transthyretin isoforms. (0.99 MB DOC) [file pone.0000756.s001.doc]

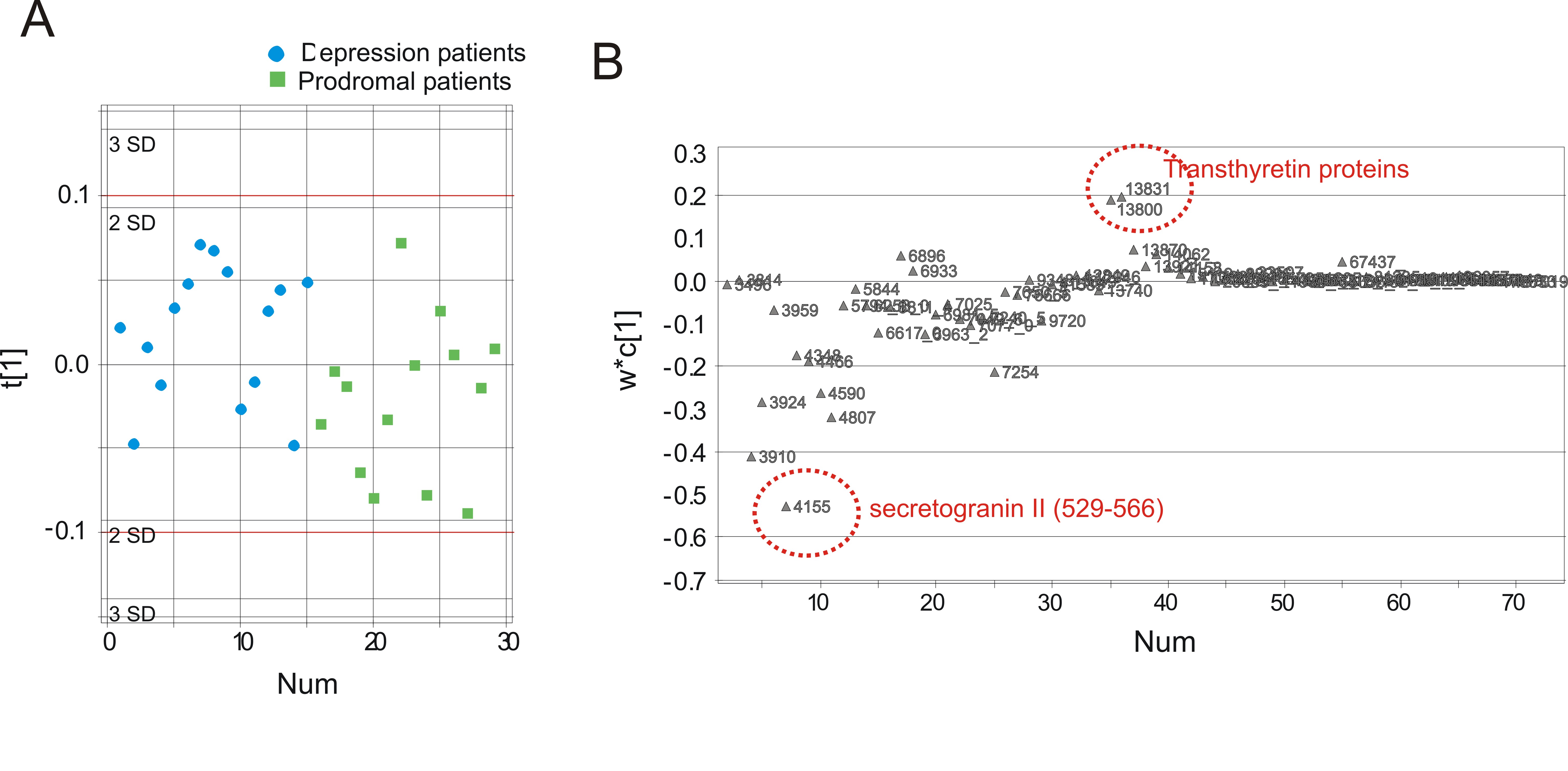


Supplementary Figure 1. Proteomic analysis of CSF samples from patients with prodromal schizophrenia, and depression patients.

(A) and (B) PLS-DA scores plots showing a degree of separation of prodromal schizophrenia patients (■) from depression patients (●) as determined by the SELDI CSF spectra. The key changing proteomic peaks are from secretogranin II (529-566) and two transthyretin isoforms.
